# Temporal changes in thiol‐oxidized plasma albumin are associated with recovery from exercise‐induced muscle damage after a marathon

**DOI:** 10.14814/phy2.70155

**Published:** 2024-12-27

**Authors:** Christopher James, Erin M. Lloyd, Peter G. Arthur

**Affiliations:** ^1^ Proteomics International Nedlands Western Australia Australia; ^2^ School of Human Sciences (Department of Anatomy, Physiology and Human Biology) The University of Western Australia Crawley Western Australia Australia; ^3^ School of Molecular Sciences The University of Western Australia Crawley Western Australia Australia

**Keywords:** dried blood spot, endurance exercise, exercise‐induced muscle damage, human muscle, oxidative stress, OxiDx, thiol‐oxidized albumin

## Abstract

Exercise‐induced muscle damage (EIMD) can affect athlete performance and is a risk factor for major muscle injury. The temporal profile of thiol‐oxidized albumin, a marker of oxidative stress, has shown potential in assessing recovery from EIMD in non‐athletically trained participants but not yet in trained participants. Our primary aim was to assess whether there are changes in the level of thiol‐oxidized albumin after a marathon in athletically trained participants. Twenty participants completed a marathon and collected daily dried blood spots from 3 days prior to and 7 days after the marathon to measure thiol‐oxidized albumin using a novel methodology (OxiDx). Participants were also assessed for indirect markers of EIMD prior to and on days 2 and 5 post‐marathon. The level of thiol‐oxidized albumin peaked at 2 days and remained elevated until 5 days after the marathon and correlated with indirect measures of EIMD. Furthermore, time of recovery for thiol‐oxidized albumin varied between participants, some recovered at 3 days post‐marathon whereas others extended beyond 7 days post marathon. Tracking temporal changes in the level of thiol‐oxidized albumin has potential to be useful in managing recovery from EIMD in athletes, particularly considering the ease of the OxiDx methodology.

## 1 INTRODUCTION

Endurance running is a popular aerobic sport well‐recognized for its beneficial effects on health and strength (Boullosa et al., 2020; Hespanhol Junior et al., 2015; Wirnitzer et al., 2022). Shorter endurance events, such as the 10 km fun run or the 5 km Parkrun are popular, mass participation events, attracting millions of global participants each year (Cushman et al., 2014; Quirk et al., 2021). Even the most challenging of running events, the marathon race (42.2 km), has an annual global participation of over 1 million (Reusser et al., 2021). Although endurance running confers health benefits, it is not without risk.

Endurance events, as well as long training runs, cause considerable stress to muscles, with exercise‐induced muscle damage (EIMD) resulting when the stress exceeds the mechanical limits of the muscle (Andrade et al., 2021; Del Coso et al., 2013; Higashihara et al., 2020; Quinn & Manley, 2012). Damage can be the consequence of a single injurious event or the result of accumulated microscopic muscle damage. There are also a number of risk factors which influence the susceptibility of a runner to muscle damage including age and previous injuries (Peake et al., 2017). Depending on the severity of damage, recovery can take a few days to several weeks (Chalchat et al., 2022; Harty et al., 2019; Peake et al., 2017).

Recovery from EIMD is complex and involves various types of inflammatory cells, including neutrophils and macrophages. Following the initial damage, there is a transitory accumulation of neutrophils from approximately 30 min up to approximatly12–24 h (Smith et al., 2008; Tidball, 2005; Toumi et al., 2006). Neutrophils are phagocytic and secrete proteases and generate the potent oxidant hypochlorous acid (Tidball, 2005; Winterbourn et al., 2016). Neutrophils also produce a range of proinflammatory cytokines and chemotactic molecules that attract macrophages to the damage site (Tidball, 2005, Toumi et al., 2006).

Macrophages are the main inflammatory cells that accumulate in damaged muscle, appearing within 24 h after damage (Bernard et al., 2022; Chazaud, 2016). Different macrophage subtypes play major roles in the early and later stages of muscle regeneration (Chazaud, 2016). Pro‐inflammatory macrophages (sometimes referred to as M1) are involved in early events of phagocytosis, remodeling of the extracellular matrix, and myogenesis with myotube formation if necrosis of myofibres has occurred (Chazaud, 2016; Martins et al., 2020; Minari & Thomatieli‐Santos, 2022). Later macrophages (M2) secrete anti‐inflammatory cytokines and are involved in maturation of new myofibres if necrosis has occurred, and for the resolution of the regenerative process (Chazaud, 2016; Yang & Hu, 2018). The time period for the presence of macrophages at the injury site is variable and dependent on the severity of the injury, with some studies indicating macrophages can persist within the muscle for up to several weeks after the initial damaging event (Bernard et al., 2022; Ziemkiewicz et al., 2021).

One of the challenges for practitioners and athletes is identifying when the muscle has recovered from EIMD, as return to competition or training prior to recovery risks further exacerbating the damage and is a risk factor for a major muscle injury, such as a muscle tear (Freckleton & Pizzari, 2013; Opar et al., 2015; Stauber, 2004). Given that recovery times are variable between individuals, decisions about returning to training and competition would benefit from knowledge about the temporal recovery from EIMD. To this end, there are few studies that have investigated the daily temporal profile of recovery from EIMD, perhaps because the technique which is considered to be the gold standard for EIMD measurement, a functional assessment of muscle strength, can be time consuming to perform and requires specialized equipment (e.g., isokinetic dynamometer) (Chalchat et al., 2022; Damas et al., 2016; Fares et al., 2022; Nosaka & Clarkson, 1995).

As part of the repair and recovery process, inflammatory cells, such as neutrophils and macrophages, generate reactive oxygen species (ROS) leading to a state of oxidative stress within the damaged muscle (Canton et al., 2021; Chazaud, 2016; Powers et al., 2011). Albumin, the predominant plasma protein, is also present in the interstitium (Ellmerer et al., 2000). Albumin moves from the blood across the capillary wall into interstitial compartments, and returns to the blood through the lymphatic system (Fanali et al., 2012). Albumin is consequently exposed to oxidants within muscle tissue and can therefore potentially be used as a biomarker of oxidative stress in muscle tissue (Al‐Mshhdani et al., 2021). Recently, we found that changes in the levels of thiol‐oxidized albumin, were persistently elevated following EIMD (James et al., 2024). In this study, non‐athletically trained participants self‐collected one drop of blood from their fingertip using dried blood spot cards each day for 10 days for the determination of the levels of thiol‐oxidized albumin (James et al., 2024). The temporal pattern of change in thiol‐oxidized albumin revealed that the effects of EIMD persisted for at least 8–10 days, in contrast to MVC which returned to pre‐EIMD levels 6 days after exercise. Thus, the sensitivity of thiol‐oxidized albumin might be useful in monitoring muscle repair following EIMD, especially given the relative simplicity of blood sample collection from the fingertip (James et al., 2024). However, our previous study utilized a dynamometer to cause EIMD with eccentric leg contractions, which may not be reflective of muscle damage caused by running (James et al., 2024).

There are multiple strands of evidence that the heightened physical demands of a marathon cause damage to muscles. Muscle biopsies from the gastrocnemius muscle provided direct evidence of muscle damage with disrupted ultrastructure and increased inflammatory cell presence (Hikida et al., 1983). Magnetic resonance imaging (MRI) demonstrated that a marathon causes damage to the hamstring muscles (Higashihara et al., 2020). The release of the intracellular proteins into the blood has also been used to assess the level of direct muscle damage caused by EIMD. The release of intracellular proteins (e.g., creatine kinase, CK) requires the permeabilisation of the muscle cell plasma membrane and generally increase within 48 h after a marathon, generally returning to pre‐marathon levels by 7 days (Bernat‐Adell et al., 2021; Kaleta‐Duss et al., 2021; Neubauer et al., 2008; Ryu et al., 2016). Unlike the release of intracellular proteins, inflammatory responses are characteristic of the repair process from muscle damage, and biomarkers of systemic inflammation, as well as those linked to neutrophils and macrophages have been shown to increase (Nosaka et al., 2002; Paulsen et al., 2012; Stozer et al., 2020; Tidball, 2005). Biomarkers of oxidative stress are also elevated following a marathon and have been attributed to both the muscle repair and adaptation process following EIMD but there is no consensus on the temporal profile of recovery to pre‐marathon levels (Briviba et al., 2005; Jamurtas, 2018; Sahlin et al., 2010; Thirupathi et al., 2020).

Marathons are muscle‐damaging in athletically trained runners, so we hypothesized that a marathon would cause an increase in the level of thiol‐oxidized albumin in the days following a marathon. To assess the daily level of thiol‐oxidized albumin for up to 7 days post marathon we employed a methodology previously described by James et al. (2024) referred to hereafter as OxiDx (James et al., 2024). To assess the extent of EIMD, we measured muscle strength and delayed onset muscle soreness (DOMS), as both are commonly used to assess muscle damage and recovery. CK was also measured as it is widely used as a blood biomarker to report muscle damage. Additionally, we examined whether there was an association between thiol‐oxidized albumin and CK, DOMS and muscle strength. A follow up study involving concentric endurance cycling in a separate group of athletes was undertaken to establish whether there was an increase in thiol‐oxidized albumin in the days after intense non‐damaging exercise.

## 2 MATERIALS AND METHODS

### 2.1 Participants

Twenty healthy male (*n* = 7) and female (*n* = 13) participants [age, 29.7 ± 4.1 years (range, 21–55 years); height (stadiometer), 182.8 ± 7.7 cm (range, 172–191 cm); weight (digital platform scale), 75.0 ± 5.8 kg (range, 75.0–92 kg)] provided their informed written consent to participate in this study. All the participants were accustomed to high‐intensity aerobic exercise, having completed at least one marathon event in the past 12 months and were running at least 40 km in a 5‐day week. All participants were enrolled to compete in the 2023 Perth Running Festival Marathon Event. The participants were screened to ensure they had no pre‐existing medical condition, no contraindication to exercise, and were not taking any medication or supplement affecting inflammation or oxidative stress. The participants were informed that they were allowed to participate in their normal daily activity but were requested to refrain from any intense physical exercise (aside from the marathon event) for the duration of the study (3 days pre‐marathon and 7 days post‐marathon). The Ethics Committee of the University of Western Australia (UWA) approved this study (approval number 2023/ET000581), and all procedures conformed to the Declaration of Helsinki.

### 2.2 Experimental design

**FIGURE 1:**
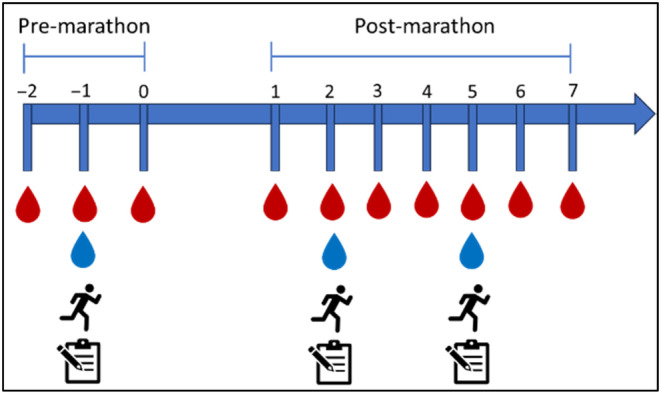
Representation of study testing schedule. Capillary blood samples (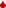) from the fingertip were collected for OxiDx analysis each day for 3 days prior to and 7 days after the completion of a marathon. Participants completed the marathon on Day 3 of the pre‐marathon testing period. Participants presented at the UWA testing laboratory on Day 2 of the pre‐marathon testing period and on days 2 and 5 of the post‐marathon testing period for a capillary blood sample for the determination of CK activity (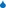), assessment of maximal voluntary contraction of the right and left knee extensors (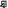) and a muscle soreness assessment (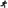).

Participants completed 3 days of pre‐marathon (no‐exercise) baseline testing to assess the normal day‐to‐day variability in the level of thiol‐oxidized albumin and determine baseline levels of EIMD markers. This 3 day ‐period was followed by undertaking the marathon event on Day 3 of the baseline testing period (Figure 1). The marathon event was then followed by a 7‐day post‐marathon testing period (Figure 1). On Day 2 of the pre‐marathon testing period, each participant presented at the UWA testing laboratory for a soreness assessment, capillary blood sample for the assessment of CK activity, and a maximal voluntary contraction (MVC) assessment of the right and left knee extensors. On days 2 and 5 post‐marathon, the participants returned to the UWA laboratory for a repeat assessment of muscle soreness, CK, and MVC. Each participant self‐collected a dried blood spot sample for OxiDx analysis of the level of thiol‐oxidized albumin each day of the study period.

Participants were also asked to record their food intake to ensure they did not consume excessive quantities of antioxidant‐rich foods. Participants were provided with a list of antioxidant‐rich foods (e.g. dark chocolate, berries and kale etc.) and were advised to either avoid these foods entirely or use their discretion regarding excessive consumption (Table 1). A review of the food journals by the research team found no abnormal consumption of antioxidant‐rich foods by participants. Additionally, participants were asked to not consume whey‐based protein supplements as the high concentration of cysteine present in these supplements might affect the level of thiol‐oxidized albumin. Finally, physical activity levels and sleeping patterns were also recorded each day to help participants reproduce these conditions for the entire study period.

**TABLE 1:** List of antioxidant‐rich foods provided to participants.

Tea and coffee (including black, green and other herbal types)
Dark chocolate
Berries (e.g., blueberries, raspberries, cranberries)
Dark leafy greens (e.g., kale, spinach)
Juices (e.g., apple, cranberry)
Herbs and spices (e.g., clove, mint, oregano)
Nuts (e.g., walnuts)

#### 2.2.1 Marathon event

The marathon race commenced at 6:30 am, and all subjects completed the entire marathon, a distance of 42.2 km (or 26.2 miles). Completion times ranged from 2 h 45 min to 5 h 32 min with an average completion time of 3 h 36 min. The course featured a majority of flat running with a maximum elevation (highest distance reached during the race) of 32 m and gain (total distance climbed during the race) of 187 m.

### 2.3 UWA Laboratory testing sessions

Prior to testing, all participants' height, weight, and age were recorded. Participants were also familiarized with the VALD DynaMo system for MVC testing and the OxiDx dried blood spot capillary blood sampling procedure for the assay of oxidative stress to ensure correct use when provided with their sample collection kit.

#### 2.3.1 Maximal voluntary contraction protocol and rate of force development

MVC of the quadriceps was performed using the VALD DynaMo System (VALD Performance, Melbourne Australia) at a 200 Hz analog‐to‐digital conversion rate. The VALD DynaMo system has previously been reported as an accurate and valid device for maximal muscle torque testing (Maly et al., 2024; Thotawatta et al., 2023). A strap was placed around the participants' ankle and connected to the load cell of the VALD DynaMo which was secured to a fixed location for consistent measurements. Participants were tested seated during an isometric knee extension at a knee joint angle of approximately 80 degrees. For each MVC test, the participant performed five static maximal voluntary knee extension efforts. They were verbally instructed to extend the knee as fast and forcefully as possible and encouraged to maintain their effort for 3 s. A 60‐s break was allowed between each attempt, consistent with other studies which have employed MVC testing (Andersen et al., 2014; Crameri et al., 2007; James et al., 2024; Yoon et al., 2008). During all trials, a visual display of the dynamometer force was provided to the participants on a computer screen. For each attempt, maximal torque was measured from the plateau region of the torque tracing curve, and the best attempt for each participant was considered to be the MVC. Rate of force development (RFD) was recorded for the repetition considered to be the MVC. Both right and left knee extensors were tested.

#### 2.3.2 Capillary blood collection

For in‐lab blood collections, a 100 μL capillary blood sample was collected from the fingertip into a microfuge tube. The blood was centrifuged immediately at 2000*g* for 3 min, and plasma was collected and divided into separate aliquots for analysis and then frozen at −80°C. For the at‐home blood collection, the participants were provided with a sampling kit that included ethanol swabs, lancets, and sample collection devices containing dried blood spot paper (PerkinElmer 226 Spot Saver RUO Card) stored in the presence of silica gel desiccant. Prior to testing, the participants were trained and familiarized with the capillary blood collection protocol. They were instructed to place two drops of blood from the finger onto the centre circle of each collection card. The cards were then stored in silica gel desiccant away from sunlight until analysis. For the duration of the study, the blood collection process was repeated each morning at least 15 min after waking and prior to any food intake.

In this study, all 200 self‐collected dried blood spot samples were suitable for analysis. Additionally, none of the participants reported any adverse issues with the self‐collection of dried blood spots performed at home (e.g., infection, excessive pain, or needlestick injury).

#### 2.3.3 Plasma creatine kinase, delayed onset muscle soreness (DOMS) measurements

Plasma CK levels were measured in duplicate using a CK‐NAC kit (Cat No: CK110, Randox Laboratories, Parramatta, NSW, Australia), and analyzed using a BioTek Powerwave XS Spectrophotometer fitted with the KC4 (V34) program (BioTek instruments). In brief, plasma was diluted in 0.1% (w/v) NaCl before being loaded into a 96‐well plate. The enzyme reagent was then added, and absorbance (340 nm) was measured every minute at 37°C. The resulting data were analyzed for the rate of absorbance change over 30 min, using only the linear period of the reaction.

Prior to exercise and/or each MVC testing, participants used a visual analogue scale to record their level of muscle soreness in their knee extensor on a 100 mm horizontal line while the center of their quadriceps muscle was palpated. On this scale, “0” indicated that there was no pain and “100” indicated the highest level of pain.

#### 2.3.4 OxiDx assay for thiol‐oxidized albumin

The level of thiol‐oxidized albumin was measured using the OxiDx technique previously described (James et al., 2024). In brief, albumin was extracted into 0.05% tween 20 in 20 mM phosphate with further binding to Cibacron Blue F3GA agarose (Cat No: C1535‐50ML). Albumin was eluted with 30 mM phosphate buffer, pH 8.2, with 2.5% (w/v) SDS pH 8.2.

Capillary electrophoresis separations of oxidized albumin (OA) and reduced albumin (RA) were performed using the Agilent 7100 CE system. A bare‐fused silica capillary with a diameter of 50 μm was purchased from Polymicro Technologies (Tucson, AZ, USA). Prior to use, the new capillary was flushed in sequence with methanol (10 min), double deionised (DDI) water (2 min), 0.1 M NaOH (15 min), DDI (2 min), and background electrolyte (20 min). Separations were performed in a capillary that was 32 cm long, with an effective length of 24 cm to the detector. The background electrolyte was a 30 mM phosphate buffer, pH 8.2, with 2.5% (w/v) SDS. Sample injection was performed hydrodynamically (10 mbar for 10 s). Running voltage was set at +12 kV with a resulting current of 61 μA. Absorbance at 200 nm was recorded. A post‐conditioning of the capillary was performed after every sample by flushing the capillary with DDI (1 minute) and a background electrolyte solution (3 min). For the ratiometric calculation of the percentage of oxidized albumin (%OA) the following formula was used:

Percentage of oxidized albumin (%OA) %OA = OA/RA × 100%.

### 2.4 Follow up study—concentric endurance cycling

Seven healthy male participants provided their informed written consent to participate in this study. Prior to testing, all participants' age, weight and height were recorded: average age, 23 ± 3 years (range, 19–31 years), height (stadiometer), 182.8 ± 7.7 cm (range, 172–191 cm); and weight (digital platform scale), 83.5 ± 4.8 kg (range, 75.0–94.6 kg). Participants undertook a V̇O_2_ peak test as described below. Participants were familiarized with the Biodex dynamometer (Biodex System 4, Biodex Medical Systems, Inc., Shirley, NY) for MVC testing.

#### 2.4.1 V̇O_2_ peak testing

The peak rate of oxygen uptake test (V̇O_2_ peak) was completed at a constant temperature of 22°C on a stationary air‐braked cycle ergometer (Evolution, Melbourne, Australia). The ergometer was fitted with a customized computer program for the determination of power output (Cyclemax, UWA, Australia). After a few minutes of cycling at a steady state, the test began at a power output of 100 W and increased by 30 W every 3 min until participants reached volitional exhaustion or until their power output could not be maintained for at least 20 s. Expired air was collected every 15 s, and analyzed using an indirect calorimetry system (TrueOne 2400, Parvo Medica Inc., Utah, USA).

#### 2.4.2 Experimental design

At least 7 days after the familiarization session, the participants returned to the laboratory and performed a concentric exercise protocol as follows. All of the participants performed 40 min of cycling at 70% of their V̇O_2_ peak. To ensure that they were achieving the desired level of physical exertion a rating of their perceived exertion (RPE) on a BORG scale was taken during the exercise. A heart rate monitor was also fitted (Polar FT, Kempele, Finland) to measure heart rate at baseline, during. and after exercise. Before and after exercise, a MVC assessment was completed. The participants remained in the laboratory (passively sitting) for the following 3 h, with a capillary blood sample collected every 30 min to investigate the transient increase in the level of thiol‐oxidized albumin which has previously been observed (Lim et al., 2020). The participants then returned to the laboratory every day for the following 3 days for the collection of a capillary blood sample to measure the levels of thiol‐oxidized albumin, CK, DOMS assessment and MVC test.

### 2.5 Statistical analyses

The statistical analyses for the level of thiol‐oxidized albumin, MVC, and RFD were performed on the percent changes from pre‐exercise baseline. Statistical analyses on DOMS and CK were performed on raw values. Significant differences between pre‐ and post‐exercise time points were determined using GraphPad Prism software (Version 10.1.0 GraphPad Software, Inc., USA). Data were analyzed using a *t*‐test or one‐way ANOVA with repeated measures followed by Dunnett's post hoc testing. The assumption of normality was checked prior to statistical analysis. Correlation analyses between the level of thiol‐oxidized albumin, MVC, RFD, DOMS, and CK were performed on the percent changes from pre‐exercise baseline using GraphPad Prism software. All data are presented as mean ± the 95% confidence interval (CI) of the population mean to better convey visually the level of interindividual variability for each of the markers investigated in our study. Significance was set at *p* < 0.05.

The level of change for thiol‐oxidized albumin that constitutes a significant difference between serial samples was calculated by determining the Reference Change Value (RCV), using coefficients of variation for analytical and individual variability calculated using the absolute level of thiol‐oxidized albumin from the pre‐marathon period. The RCV represents a value, for an associated probability, to account for both analytical imprecision and within‐subject biological variation (Fraser, 2011). Bi‐directional RCVs were calculated using Microsoft Excel software as described by Lund et al. (2015).

## 3 RESULTS

**FIGURE 2:**
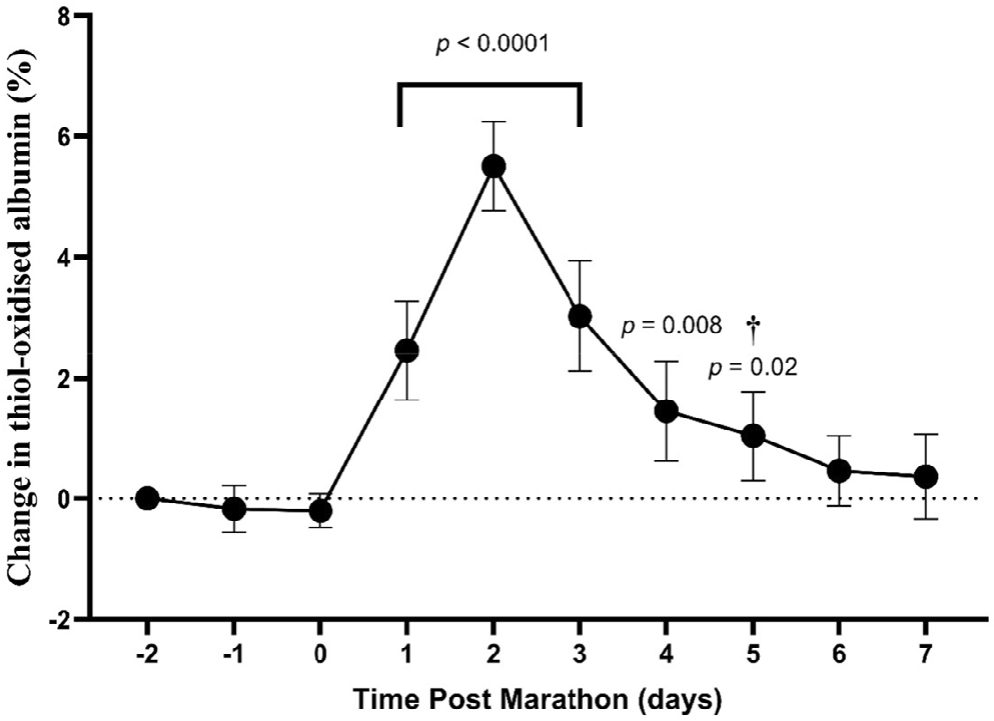
Change in the level of thiol‐oxidized albumin for the pre‐marathon and post‐marathon testing periods. Days before 0 represent the pre‐marathon testing period and day 0 represents the day of the marathon, with the marathon undertaken after testing. Data are expressed relative to the pre‐marathon baseline value of 0% at Day 0 and are means ±95% CI. † Significantly different from Day 2. *n* = 20 participants.

To test whether the level of thiol‐oxidized albumin was elevated after a marathon, blood samples were collected prior to the race (days −2, −1 and 0, when athletes were resting), and then daily for 7 days after the marathon. The absolute level of thiol‐oxidized albumin before the marathon (Day 0) ranged from 20.5% to 31.8%. To account for observed inter‐individual variability, changes in the level of thiol‐oxidized post‐marathon albumin were expressed relative to pre‐marathon baseline values at Day 0. From Day 0 baseline, there was an increase in thiol‐oxidized albumin of Δ2.45% at 1‐day post‐marathon (*p* < 0.0001), which remained elevated from Day 2 (*p* < 0.0001) until day 5 post‐marathon (*p* = 0.2, Figure 2). Thiol‐oxidized albumin peaked at 2 days post‐marathon with a change from baseline of Δ5.5% (Figure 2).

**FIGURE 3:**
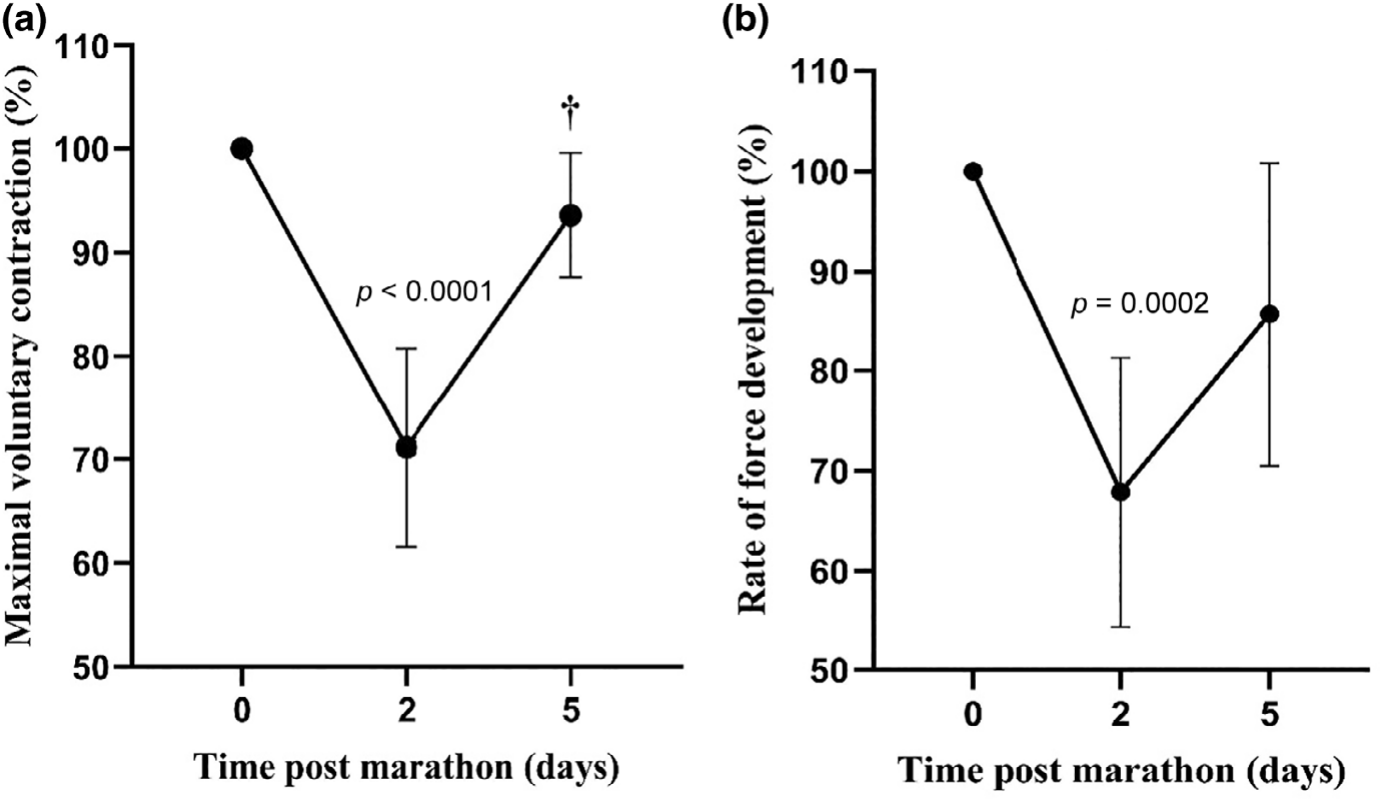
Changes in (a) maximal voluntary contraction (MVC) torque and (b) rate of force development (RFD). Data are expressed as means ±95% CI and relative to pre‐marathon values for both MVC and RFD (100%). † Significantly different from Day 2. *n* = 20 participants.

**FIGURE 4:**
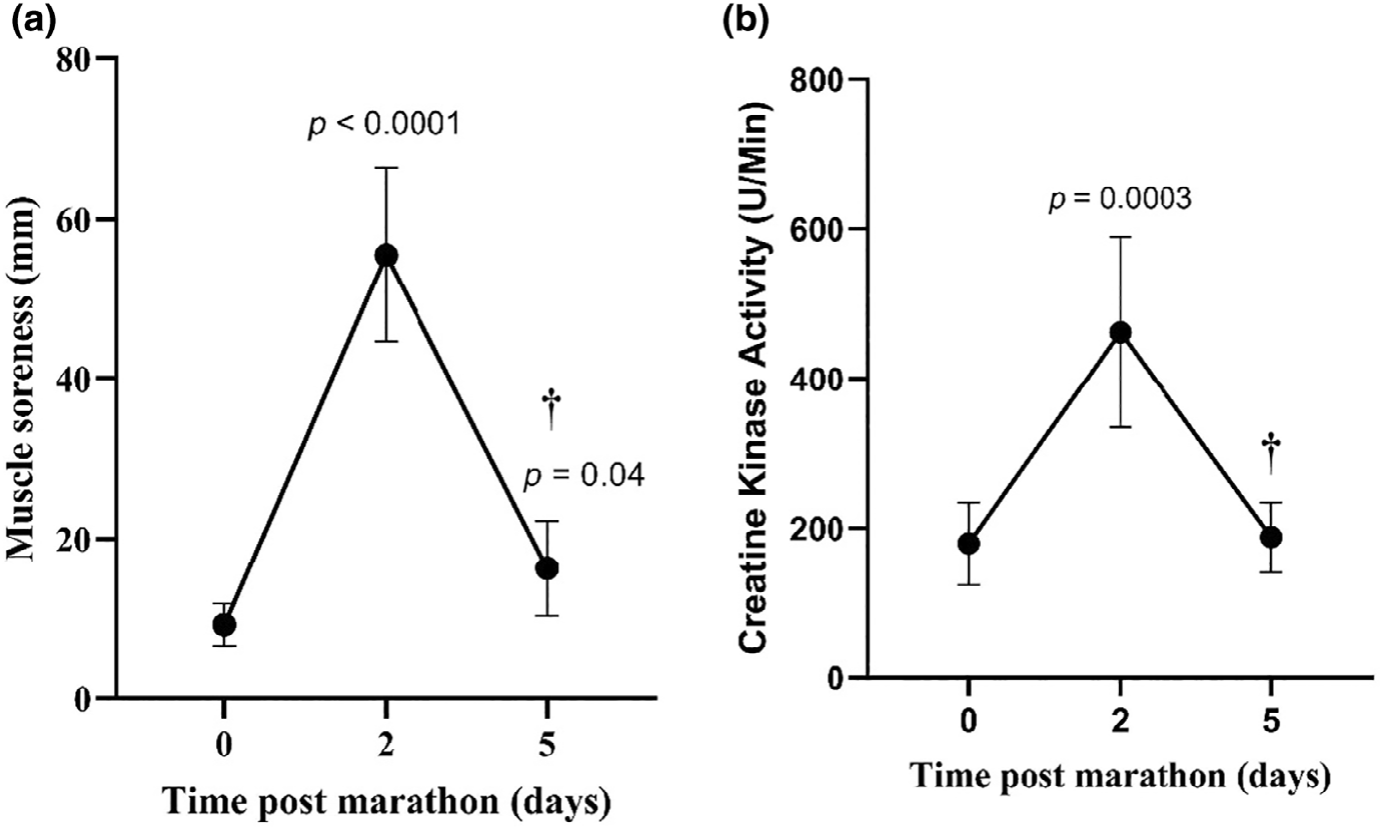
Absolute changes (a) perceived muscle soreness and (b) creatine kinase activity. Data are expressed as means ±95% CI. † Significantly different from Day 2. *n* = 20 participants.

Maximal voluntary contraction (MVC) and rate of force development (RFD) were used to determine the extent of muscle damage resulting from the marathon. MVC and RFD decreased, respectively, by 29% and 33% at 2 days post‐marathon (*p* = 0.0001), with evidence of recovery at day 5 as MVC was significantly higher at day 5 post‐marathon compared to day 2 (*p* = 0.001; Figure 3a,b). Furthermore, the significant increase DOMS (*p* < 0.0001) and CK (*p* = 0.0003) on day 2 post‐marathon is further evidence that there was muscle damage after the marathon (Figure 4a,b). By day 5, there was evidence of recovery with both DOMS and CK significantly lower than day 2 (*p* = 0.0001; Figure 4a,b). Changes in thiol‐oxidized albumin were significantly correlated with MVC (*R* ^2^ = 0.31), RFD (*R* ^2^ = 0.21), CK (*R* ^2^ = 0.35), and DOMS (*R* ^2^ = 0.51) at 2‐ and 5‐days post‐marathon (*p* < 0.01). There was no correlation between marathon finishing times and the level of thiol‐oxidized albumin, MVC, RFD, DOMS or CK activity.

A population‐based analysis does not provide information about variability in temporal changes for individual participants. Therefore, reference change values (RCV) were used to track changes in the level of thiol‐oxidized albumin for individual participants after the marathon. Estimating RCV involved calculating within‐subject variation (CVi) and the analytical coefficient of variation (CVa). The CVi was calculated from three blood samples collected on consecutive days prior to the marathon and was calculated at 1.95%. The CVa was calculated at 1.7%. A fractional increase in RCV of 1.054 was used to establish whether there was a significant (*p* < 0.05) increase the level of thiol‐oxidized albumin from the pre‐race value.

**FIGURE 5:**
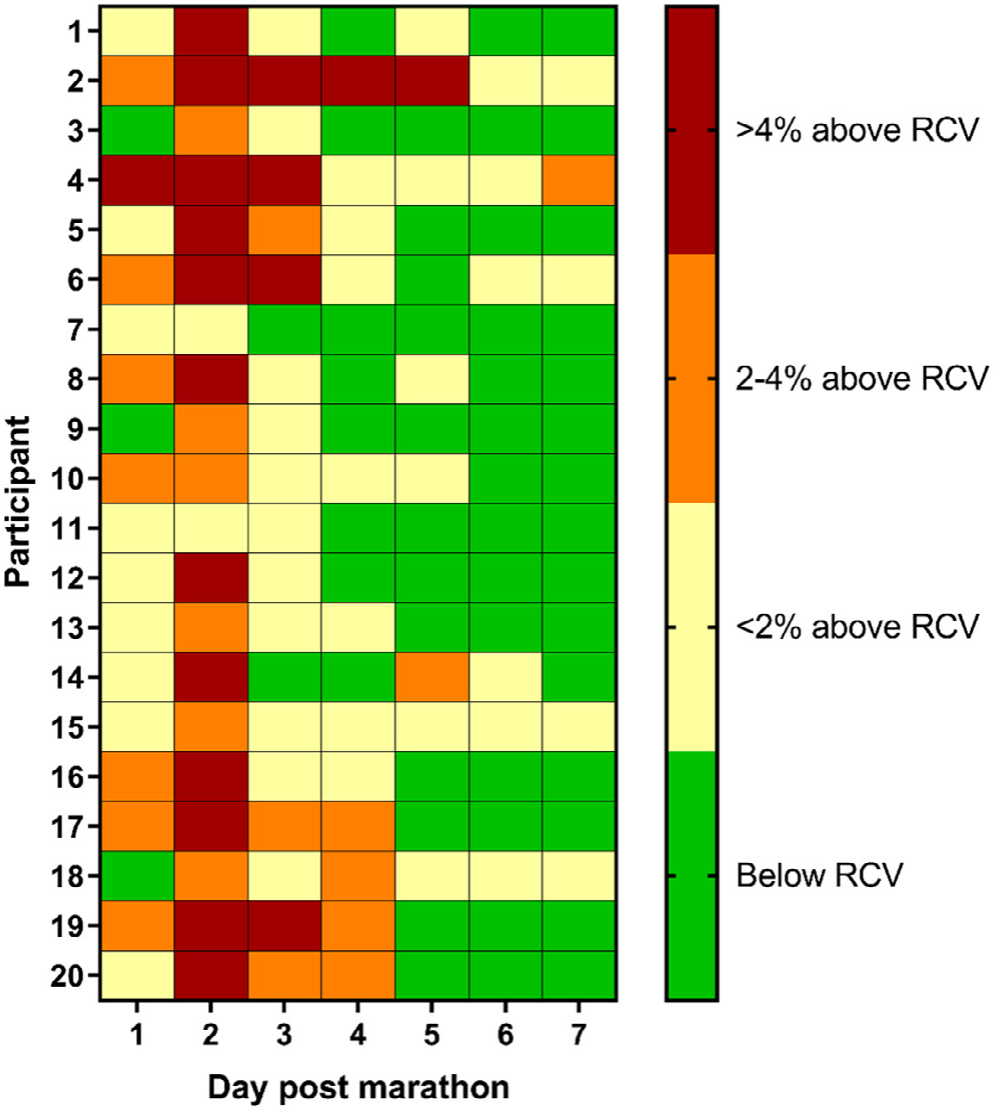
Heatmap depicting levels of thiol‐oxidized albumin that are below the reference change value or above the reference change value for 20 participants using a confidence of 95% each day after the marathon.

The level of thiol‐oxidized albumin was above the RCV cut‐off for all participants at 95% confidence 2 days after the race (Figure 5). There was considerable variability in the time of recovery between participants, with 8 participants below RCV by Day 4, but 5 participants still above RCV by Day 7 (Figure 5). To convey the individual differences in the level of thiol‐oxidized albumin following a marathon, three arbitrary categories for the level of thiol‐oxidized albumin above the RCV value were selected (Figure 5).

**FIGURE 6:**
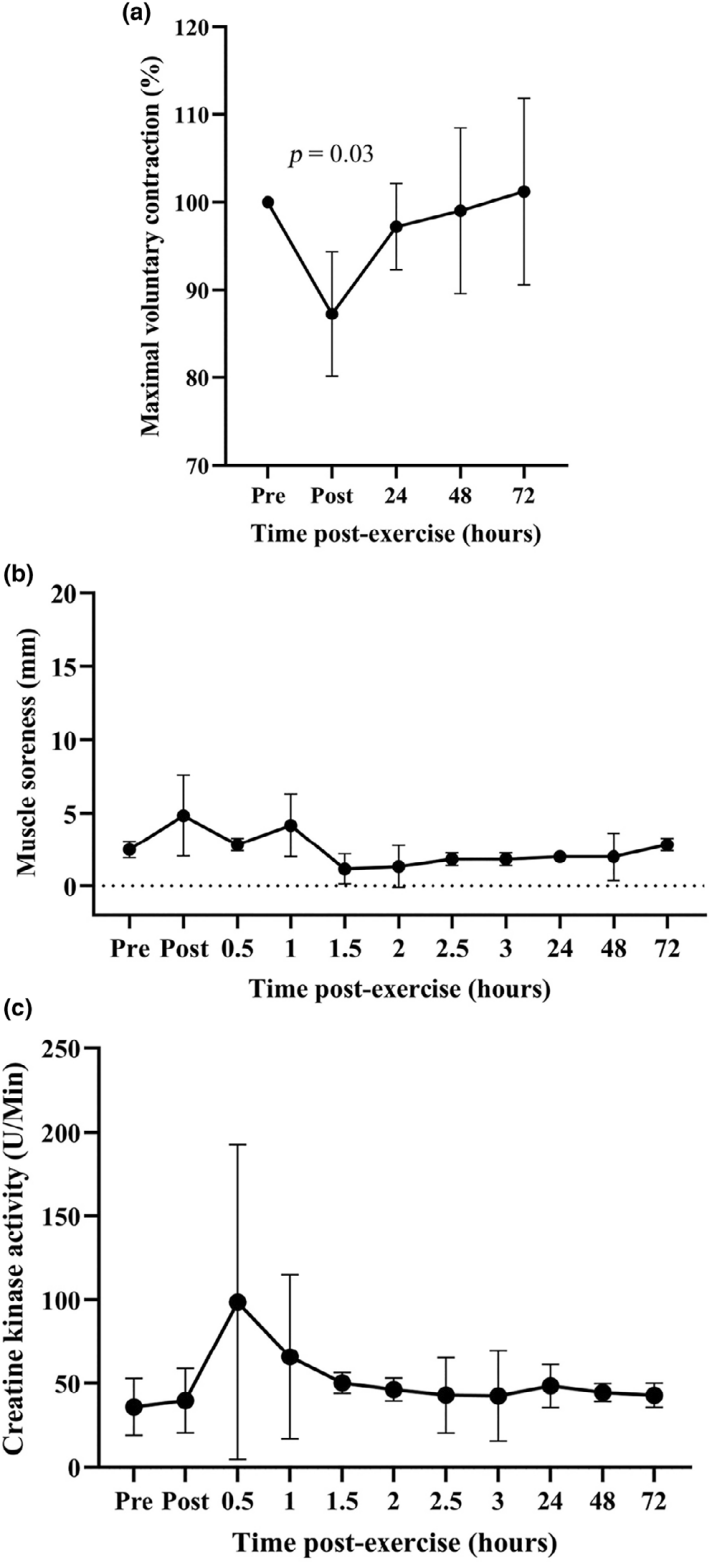
Changes in (a) maximal voluntary contraction torque after concentric exercise, (b) DOMS and (c) the activity of creatine kinase. Data are expressed as means ±95% CI and relative to pre‐exercise values for MVC (100%) and in absolute terms for DOMS and creatine kinase. *n* = 7 participants.

**FIGURE 7:**
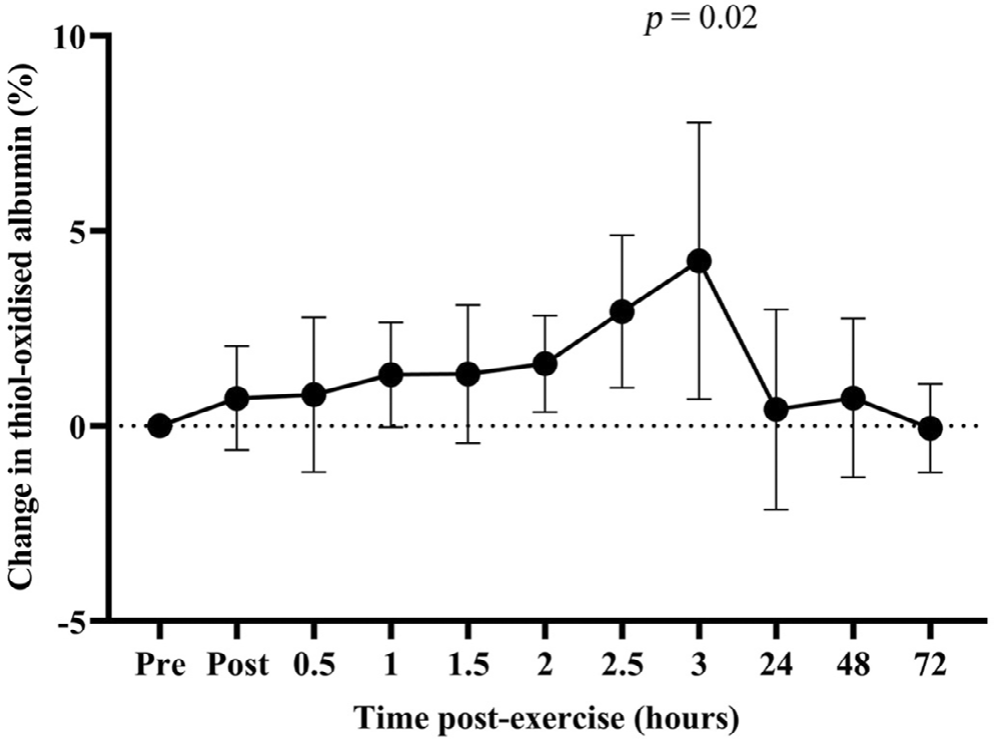
Changes in the level of thiol‐oxidized albumin. Data are expressed relative to pre‐exercise baseline values and as means ±95% CI. *n* = 7 participants.

Concentric endurance cycling exercise was undertaken to assess whether non‐damaging exercise also caused an increase in thiol‐oxidized albumin. Concentric endurance cycling exercise resulted in a significant fall in MVC (*p* = 0.03) which returned to pre‐exercise level within 1 day after exercise and remained stable over the following 2 days (Figure 6a). Concentric exercise did not cause any change in the level of DOMS or CK (Figure 6b,c). The level of thiol‐oxidized albumin was higher at 180 min after exercise than at baseline (*p* = 0.02), but returned to pre‐exercise level within 14 h, and remained unchanged for up to 72 h after exercise (Figure 7).

## 4 DISCUSSION

The primary finding of this work was an increase in the level of thiol‐oxidized albumin measured for all participants after a marathon. Consistent with our hypothesis, the level of thiol‐oxidized albumin increased and peaked 2 days after the marathon and remained elevated until 5 days after the marathon. The increase in thiol‐oxidized albumin was likely a consequence of EIMD, as changes in measures of EIMD were correlated with changes in the level of thiol‐oxidized albumin. Of note, recovery as determined by RCV was not consistent between participants, with some participants recovering by Day 3 post‐marathon and recovery for others extending beyond 7 days.

EIMD is attributed to overstretched sarcomeres and damaged extracellular matrix in skeletal muscle (Harty et al., 2019; Peake et al., 2017). To assess EIMD after the marathon, we used previously described measures: muscle force generation (MVC and RFD), muscle cell membrane permeability (CK), and perceived increase in soreness (DOMS) (Peake et al., 2017). The significant decrease in MVC and RFD at 2 days post‐marathon is evidence of EIMD. The timeline for decreased muscle force observed in this study is consistent with previous work including muscle functional tests (e.g., peak torque, counter movement jump) where there is a maximal decrement immediately post‐marathon and can remain below pre‐marathon levels at 2 days post‐marathon (Andrade et al., 2021; Higashihara et al., 2020; Petersen et al., 2007; Quinn & Manley, 2012). The release of the intracellular protein CK into the blood has also been used to assess muscle damage after a marathon, and by this measure and consistent with previous work, EIMD was evident 2 days post‐marathon (Bernat‐Adell et al., 2021; Brancaccio et al., 2007; Kaleta‐Duss et al., 2021; Kobayashi et al., 2005). The third measure of EIMD was DOMS, and by this measure, EIMD was also evident at 2 days post‐marathon. The etiology of DOMS is uncertain, but several authors have provided evidence that changes in DOMS are related more to inflammation in the extracellular matrix rather than directly with damaged myofibres, as is the case with CK (Mizumura & Taguchi, 2016; Nosaka et al., 2002; Paulsen et al., 2010; Peake et al., 2017).

In our previous work which also utilized OxiDx methodology, thiol‐oxidized albumin reached peak levels 2 days after muscle damage caused by eccentric exercise of the knee extensors in non‐athletically trained participants (James et al., 2024). However, as this was a laboratory‐based study with untrained participants, it was uncertain as to the extent to which the temporal changes in thiol‐oxidized albumin applied to trained athletes. The current study supports the earlier eccentric exercise work, albeit with some differences. In both studies, thiol‐oxidized albumin peaked at 48 h post‐exercise, although here, thiol‐oxidized albumin was elevated 1 day post‐marathon, but not for eccentric exercise (James et al., 2024). The peak observed at 48 h in both studies is not consistent with direct damage to myofibres and loss of muscle force, as these mechanisms would be expected to result in an increase in ROS generation immediately following or within 24 h after exercise (James et al., 2024).

Recovery from EIMD is dependent on the extent of the injury and the individual's susceptibility to EIMD, lasting between several days and up to several weeks for severe EIMD (Harty et al., 2019; Owens et al., 2019; Peake et al., 2017; Stozer et al., 2020). In this study, thiol‐oxidized albumin was not significantly elevated on Day 6 post‐marathon, whereas thiol‐oxidized albumin was still significantly elevated at the conclusion of our previous investigation, 10 days post‐eccentric exercise of the knee extensors (James et al., 2024). The most likely explanation for these observed differences is the difference in study populations, namely non‐athletically trained participants in our previous eccentric exercise study and athletically trained participants in the present study. Running is a movement that features eccentric muscle contractions, and there is extensive evidence for the long‐lasting protective effect of prior eccentric contractions upon skeletal muscle in subsequent bouts of exercise (referred to as the repeated bout effect) (Chen & Nosaka, 2006; Lima & Denadai, 2015; Margaritelis et al., 2015; Nikolaidis et al., 2007; Nosaka & Clarkson, 1995). Therefore, the trained athletes in this study might have been protected from some EIMD while the previous cohort of untrained participants were not, resulting in more substantial EIMD and the longer timeline for recovery observed in the previous study. Another possible explanation is that eccentric exercise of the knee extensors caused more substantial localized muscle damage compared to the marathon, and as a consequence, additional time was required for recovery from muscle damage. However, further work is needed to explore this concept as there are several alternative reasons why there was a temporal difference between eccentric exercise and marathon including type of exercise (eccentric versus concentric), involvement of different muscle groups (calves, quadriceps, hamstring etc.), and duration of exercise.

Consistent with the concept that the level of thiol‐oxidized albumin tracks the level of muscle damage, high‐intensity endurance cycling, a non‐damaging form of exercise, did not result in any evidence of long‐lasting muscle damage and no long‐lasting elevation of thiol‐oxidized albumin. The level of thiol‐oxidized albumin was only elevated at 180 min post‐exercise, returning to baseline by 24 h after exercise. Comparable results were obtained in two prior studies, including the study of Lim et al. (2020), who adopted a concentric exercise protocol that was identical to that in this study, and that of Lamprecht et al. (2008) in which participants cycled for 40 min at 70, 75 and 80% of V̇O_2_max (Lamprecht et al., 2008; Lim et al., 2020). The participants in the study of Lamprecht et al. (2008) had their blood collected before and immediately after exercise as well as 30 min and 30 h after exercise (Lamprecht et al., 2008). They found that the thiol oxidation state of albumin was higher than baseline 30 min after all three exercise intensities but returned to pre‐exercise values by 30 h (Lamprecht et al., 2008).

The elevated level of thiol‐oxidized albumin at 180 min post‐concentric exercise in this study may be related to muscular fatigue and not muscle damage. In support of this interpretation, MVC was lower immediately post‐exercise, but had recovered by 24 h after exercise, with no other measure of EIMD being elevated in the days following concentric exercise. One possible source of the ROS responsible for the increase in the thiol oxidation state of albumin might be the increase in the population of leukocytes recruited to the muscle after exhaustive exercise. Indeed, exhaustive exercise has been reported to cause an increase in peripheral blood leukocytes numbers within 30–180 min after exercise, which return to baseline by 24 h post‐exercise (Abbasi et al., 2014).

A potential source of ROS responsible for the peak in the level of thiol‐oxidized albumin at 48 h post‐marathon are inflammatory cells associated with repair pathways (e.g. neutrophils and macrophages), which have been shown to generate a range of ROS (Deyhle & Hyldahl, 2018; Peake et al., 2017; Smith et al., 2008; Tidball, 2005; Toumi et al., 2006). Neutrophils reach their peak concentration between 1 and 6 h after a damaging bout of exercise, and then rapidly decline to pre‐exercise values by approximately 24 h post‐EIMD (Peake et al., 2017; Tidball, 2005; Torres‐Ruiz et al., 2023). Therefore, it is unlikely neutrophils are responsible for the level of thiol‐oxidized albumin peaking at 48 h. Phagocytic macrophages, known as M1 macrophages, reach peak concentrations at approximately 48 h post‐injury, while non‐phagocytic macrophages (M2 macrophages) can remain at elevated levels in the injured muscle for several days afterwards, depending on the extent of the injury (Bernard et al., 2022; Chazaud, 2016; Martins et al., 2020; Wang & Zhou, 2022). The presence of macrophages from 24 h onward in the damaged muscles indicates that macrophage‐derived ROS could be responsible for increasing the level of thiol‐oxidized albumin in the days following EIMD. If so, then the increase in the level of thiol‐oxidized albumin after EIMD would reflect the activity of macrophages and muscle repair mechanisms rather than direct damage to the myofibres. Further work will be needed to test this proposition.

Given that increased levels of thiol‐oxidized albumin are likely to be linked to the inflammatory repair response, rather than directly with myofibre damage and muscle force loss, monitoring the level of thiol‐oxidized albumin may be practically useful in monitoring recovery from EIMD caused by training or competition. Training or competing without adequate recovery from EIMD can impact running performance (Burt et al., 2023; McMahon et al., 2021; Quinn & Manley, 2012). For example, in a study by Marcora and Bosio (2007), EIMD significantly reduced running speed and affected self‐paced time trial performance (Marcora & Bosio, 2007). Chen and Nosaka (2006) found increases in heart rate, minute ventilation, respiratory exchange ratio, ratings of perceived exertion, blood lactate concentration, and stride frequency, as well as reductions in stride length and range of motion of the ankle and knee in participants with EIMD (Chen et al., 2007). These data suggest that changes in compromised muscle function due to muscle damage contribute to the reduction in running economy. In addition, training or competing without adequate recovery increases the risk of sustaining a serious muscle injury (i.e., muscle tear). For example, 18.2% of runners self‐reported at least one new lower‐extremity injury due to marathon participation (Van Middelkoop et al., 2008).

Monitoring recovery from EIMD would benefit athletes, but existing measures such as CK and DOMS lack sensitivity. In this context, there is extensive literature documenting inter‐subject variability in CK (attributed to age, sex, race, and muscle mass) and DOMS (attributed to the subjective nature of pain) (Brancaccio et al., 2007; Koch et al., 2014; Nosaka et al., 2002; Nosaka & Clarkson, 1996; Sayers & Clarkson, 2003). One approach to improve sensitivity to detecting EIMD is to use reference change value (RCV), an approach that is particularly useful when within‐subject variation is much smaller than between‐subject variation (Fraser, 2011). However, it is notable that most studies do not employ RCV to account for between subject variation when investigating changes in CK and DOMS. Using RCV for the level thiol‐oxidized albumin, we found there were differences in the temporal recovery between individual participants. Thiol‐oxidized albumin was elevated in all marathon participants at Day 2 post‐marathon, which indicates that EIMD was present in all participants. By Day 7, 15 runners had fallen below the RCV cutoff (95% confidence), but 5 runners were still elevated above the cutoff value, which indicates variability in recovery times from the marathon. Given this variability in recovery between runners, there is potential for runners to recommence training with damaged muscles if only grouped observations, which indicated recovery by Day 6 post marathon, were used to determine recovery from EIMD.

The reason(s) for the temporal variability in recovery of thiol‐oxidized albumin between runners was not investigated in this study. One possibility is physical fitness, as higher levels of fitness have been associated with a shorter time to recovery from exercise induced oxidative stress (Lu et al., 2021). The range in completion times (from 2 h 45 min to 5 h 32 min) suggests some variability in physical fitness among our participants. These differences could affect the extent of muscle damage from the marathon and therefore the temporal profile of the level of thiol‐oxidized albumin post marathon. However, we found no correlation between completion time and peak thiol‐oxidized albumin or day of recovery (not reported). Further work with additional measures of physical fitness (e.g., VO_2_max) will be needed to test this possibility.

The temporal variability in recovery of thiol‐oxidized albumin between runners may reflect the extent of muscle damage incurred by the marathon. This concept supported by significant correlations of thiol‐oxidized albumin with CK, DOMS and muscle strength as measures of muscle damage. However, a limitation of this study was that we were not able to investigate temporal relationships because we did not assess day‐to‐day changes in CK, and muscle strength prior to, and following the marathon race. The reason for the lack of day‐to‐day measures of CK and muscle strength were logistical, as participants would have been required to attend the laboratory. In contrast, thiol‐oxidized albumin could be readily monitored daily because participants could collect blood samples at home.

## 5 PERSPECTIVE

Our data provide evidence that the level of thiol‐oxidized albumin may provide a useful blood biomarker of repair following EIMD. Of note, the validity of this interpretation awaits the use of muscle biopsies in future studies to test if the changes in the level of thiol‐oxidized albumin tracks closely with muscle repair. The utility of using thiol‐oxidized albumin to monitor recovery from EIMD would also benefit from a better understanding of the sources of ROS causing albumin oxidation. If such work was to corroborate our interpretations, the use of RCV for the level of thiol‐oxidized albumin as an indirect blood marker of muscle repair could be helpful in managing recovery and return to training or competition in athletes following a bout of damaging exercise or a muscle injury. The measurement of thiol‐oxidized albumin is particularly useful considering the ease of the OxiDx methodology which permits the collection of non‐invasive serial small blood samples from the fingertips. The ability to track temporal changes in thiol‐oxidized albumin could also be useful in improving our understanding of the role of ROS in other aspects of exercise, training, and competition where oxidative stress is evident.

## AUTHOR CONTRIBUTIONS

All authors contributed to the study conception and design. Material preparation, data collection, and analysis were performed by CJ, PA and EL. The first draft of the manuscript was written by CJ, PA. All authors commented on subsequent versions of the manuscript. All authors read and approved the final manuscript.

## FUNDING INFORMATION

Proteomics International provided funding for the project. No other funding was received to assist with the preparation of this manuscript.

## CONFLICT OF INTEREST STATEMENT

The authors declare no conflicts of interest.

## ETHICS STATEMENT

The Ethics Committee of the University of Western Australia approved this study (approval number 2023/ET000581), and all procedures conformed to the Declaration of Helsinki.

## Data Availability

The data supporting the conclusions of this article can be made available by the authors, upon request.
